# Bioprinting in Tissue Repair and Its ENT Applications

**DOI:** 10.3390/polym18070821

**Published:** 2026-03-27

**Authors:** Tania Vlad, Mihai Mituletu, Corina Flangea, Cristina Doriana Marina, Marioara Nicoleta Caraba, Nicolae Constantin Balica, Cristian Sebastian Vlad, Roxana Popescu

**Affiliations:** 1 Doctoral School, Faculty of Medicine, “Victor Babeș” University of Medicine and Pharmacy, 2nd Eftimie Murgu Square, 300041 Timisoara, Romania; tania.vlad@umft.ro (T.V.); cristina.marina@umft.ro (C.D.M.); 2 ANAPATMOL Research Center, Department of Cell and Molecular Biology, Faculty of Medicine, “Victor Babeș” University of Medicine and Pharmacy, 2nd Eftimie Murgu Square, 300041 Timisoara, Romania; mihai.mituletu@umft.ro (M.M.); popescu.roxana@umft.ro (R.P.); 3 Timisoara Municipal Clinical Emergency Hospital, Clinic of ENT, Revolutiei Blvd. 6, 300041 Timisoara, Romania; balica@umft.ro; 4 Department of Biochemistry and Pharmacology, Faculty of Medicine, “Victor Babeș” University of Medicine and Pharmacy, 2nd Eftimie Murgu Square, 300041 Timisoara, Romania; vlad.cristian@umft.ro; 5 ”Pius Brinzeu” County Emergency Hospital, Liviu Rebreanu Blvd 156, 300723 Timisoara, Romania; 6 Surgery IX Department, Clinic of ENT, Faculty of Medicine, “Victor Babeș” University of Medicine and Pharmacy, 2nd Eftimie Murgu Square, 300041 Timisoara, Romania

**Keywords:** biomaterials, 3D/4D bioprinting, bioinks, otolaryngology, tissue regeneration

## Abstract

Biotissues represent a new technology in tissue regeneration in otolaryngology. Various biomaterials functioning in different combinations are used as bioinks for 3D bioprinting of tissues/tissue fragments. The scaffolds can be populated with several cell categories, each offering distinct advantages and disadvantages, depending on the targeted pathology. Results from in vitro and in vivo studies on animal models are promising, with superior therapeutic potential. The combination of these elements provides promising results, enabling their potential application in personalized medicine. Based on these findings, their application in ENT (ear, nose, and throat) pathology is starting to gain traction. Despite being an emerging field, 3D/4D bioprinting in otolaryngology is rapidly evolving, increasingly replacing conventional inert materials with more sophisticated, bio-integrated alternatives. These alternatives are based on novel bioink formulation involving cells capable of proliferating and integrating the new neo-fragment organ into the host’s endogenous tissues. In this context, this review outlines novel applications that could enhance traditional procedures in ENT reconstructive medicine. Furthermore, biomimetic scaffolds for otolaryngology can be tailored to address factors influencing implant fate during the procedure and in the early and late postoperative periods.

## 1. Introduction

Rapid technological progress, an evolving patient population, changing trends in disease burden, and the implementation of health policies have all had a considerable impact on ear, nose, and throat (ENT) therapeutic management over the last few decades [1]. Historically, there has been a remarkable evolution from the traditional ENT specialist of the 1970s, focused on tonsillectomies and adenoidectomies, to the contemporary surgeon who addresses a vast spectrum of complex and diverse pathologies [2]. Despite significant advancements in surgical techniques, recent studies highlight several disorders that carry a high recurrence risk. These include cholesteatoma (with rates ranging from 3% to 30%), head and neck squamous cell carcinoma (where the 5-year survival rate remains below 50%), chronic rhinosinusitis, and benign vocal fold lesions [3,4,5,6]. In the medical field, the potential applications of biomaterials have attracted particular attention, even generating a notable trend. According to the National Institutes of Health, biomaterials are defined as compounds other than pharmacological agents, including a wide range of materials of natural or synthetic origin. These materials have applicability in replacing tissues and organs and restoring the physiological functions [7]. In contemporary medicine, biomaterials can be used in a vast array of applications, including prosthetic joints, bone grafts, dental implants, cardiovascular stents, implants for plastic, trauma, and reconstructive surgery, as well as surgical instruments [8]. Innovative biomaterials are increasingly integrated into the ENT landscape. Substantial evidence supports the use of tissue engineering for nasal, auricular, laryngotracheal, and facial bone reconstruction [8,9]. Nowadays, the repair of damaged and degenerated tissues, as well as the management of organ failure, presents a significant clinical challenge that profoundly impacts the patient’squality of life [10]. Although autografting and allografting remain the standard of care, they have a series of limitations, such as donor tissue shortages and the risk of graft rejection. Consequently, tissue engineering has emerged as an alternative through the development of biocompatible biomaterials [10]. International research efforts have demonstrated that tissue engineering represents a feasible alternative to traditional approaches in several clinical cases, including laryngeal tissue repair, tracheal reconstruction, empty nose syndrome, and nasal and septal surgery [11,12,13,14]. Furthermore, it is essential to highlight three-dimensional (3D) printing, an additive manufacturing technique with tremendous potential in otolaryngology and other medical specialties [15]. Three-dimensional bioprinting is a subset of traditional 3D printing that may overcome the conventional methods, offering a promising alternative in reconstructive surgery of the head and neck. This is achieved through the fabrication of musculoskeletal tissue, such as bone and cartilage [15]. Moreover, in recent decades, stem cells have gained attention due to their potential to restore the functionality of damaged tissues. Consequently, they are being intensively researched for applications in vocal fold regeneration alongside auricular cartilage regeneration [16,17]. The application of these new directions in reconstructive medicine is expected to yield a positive impact, facilitating a personalized approach tailored to each specific case.

The new challenges generated by 3D/4D bioprinting in regenerative medicine require in-depth expertise to develop tailored solutions for each patient. In this review, we highlighted novel biomaterials and their various combinations currently studied in tissue regeneration technology. We have expanded our analysis to include several cell types involved, their therapeutic potential, and medical applications for patients in otolaryngology.

## 2. Biomaterials Used in ENT Tissue Regeneration

Biomaterials represent a modern approach to tissue regeneration, a field that is constantly evolving. These biomaterials function not only as structural scaffolds but also provide functional support through their adaptability, bioactivity, and interconnection [18]. These biomaterials are composed of molecular structures designed to meet these requirements: carbohydrate homopolymers, carbohydrate heteropolymers, proteins and peptides, and other biopolymers.

### 2.1. Carbohydrate Homopolymers

Polysaccharides are generally recognized for their superior biocompatibility; however, these molecules present challenges regarding extraction and purification [19]. Primary research focuses on polymers abundant in nature, formed by glycosidic linkages between α- or β-anomers of glucose, such as starch or cellulose.

#### 2.1.1. Cellulose

**Figure 1:**
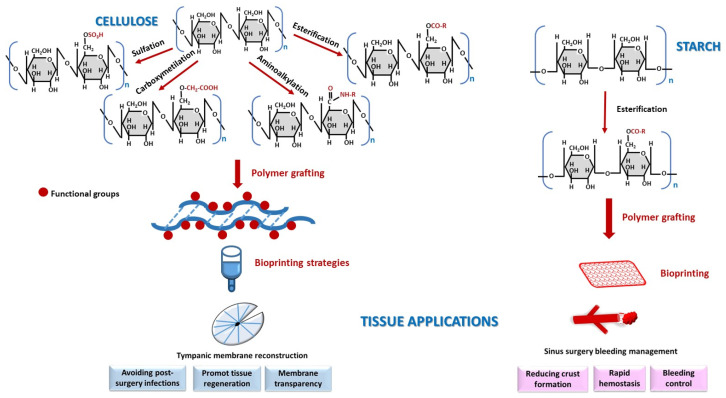
Structure and role of carbohydrate homopolymers as ENT biomaterials: cellulose, composed of repeating β-glucose linked β(1-4) units, and starch consisting of α(1-4) linked α-glucose chains.

Cellulose was among the first biomaterials used in medicine, with its clinical application recorded since 1943 [20]. Cellulose is composed of repeating β-glucose units linked by β(1-4) glycosidic bonds (Figure 1) and can serve as a scaffold for various materials in tissue regeneration [21,22,23,24,25]. Due to its molecular structure, hydroxyl (-OH) moieties are available for various interactions and the attachment of functional groups: aminoalkyl [23,26], acetate [27], alkyl, hydroxyalkyl [22,28,29,30], carboxyalkyl [28,29,30], sulfate [26], and other linkages used in hydrogel formation [29,30,31,32]. Among the alkyl and hydroxyalkyl derivatives, the hydroxylated groups exhibit higher hydrophilicity and a greater interaction capacity. A comparative analysis revealed that hydroxyethyl cellulose is more suitable for bioprinting than methylcellulose due to superior solubility and ease of functionalization [33].

Cellulose scaffold biomaterials are used in the treatment of tympanic membrane (TM) perforations and have recently been reported in the literature [34,35]. A further application has been documented in nasal cartilage reconstruction, utilizing nanofibers derived from cellulose-alginate [15,36]. Moreover, bacterial nanocellulose is a highly biocompatible material that has demonstrated an enhanced capacity for promoting chondrocyte proliferation and adhesion [8,12]. Some myringoplasty procedures have successfully used bacterial cellulose without granulation tissue formation, avoiding postoperative infection [37]. Bacterial cellulose generally offers several advantages, such as membrane transparency, which is the ability to promote tissue regeneration across all three layers of the TM [35,38,39]. This material shows great promise in reconstructive surgery of auricular cartilage [8], nasal septum [40], and trachea [12]. Despite its relatively simple molecular structure, cellulose remains a robust and reliable material for advanced reconstructive techniques in ENT.

#### 2.1.2. Starch

Starch represents another biopolymer used as a scaffold biomaterial. Its monomeric repeating unit consists of α(1-4) linked α-glucose anomers with α(1-6) branching points occurring in the amylopectin fraction (Figure 1) [41,42,43]. The presence of -OH groups facilitate forming different esters and ethers, enables enzymatic hydrolysis of the glycosidic bond, and allows the generation of mixtures with other natural or synthetic biopolymers [42]. Starch belongs to the absorbable biomaterials and can be used in sinus surgeries for the management of chronic rhinosinusitis treatment [44] as well as for postoperative hemorrhage control in other ENT procedures [45]. Compared with carboxymethylcellulose, starch-derived materials are superior in terms of reducing crust formation and the need for debridement [46]. In sinus surgery, microporous polysaccharide microspheres containing starch (Arista^TM^ (Santa Clara, CA, USA)) provide effective bleeding control and rapid hemostasis within 30–45 s of application [47]. Despite these applications, its current use remains relatively limited.

Cellulose and starch are well-established biomaterials with an extensive history of use. Currently, due to their uncomplicated molecular architecture, they serve as biodegradable scaffolds that remain in current ENT reconstructive practice.

### 2.2. Carbohydrate Heteropolymers

Carbohydrate heteropolymers are macromolecules composed of a repeating disaccharide or polysaccharide unit that exhibit structural heterogeneity (sulfation, phosphorylation, acetylation, etc.) on various units. Given their high structural diversity, these macromolecules offer significant potential for chemical manipulation to enhance their properties as biomaterials.

#### 2.2.1. Glycosaminoglycans (GAGs)

**Figure 2:**
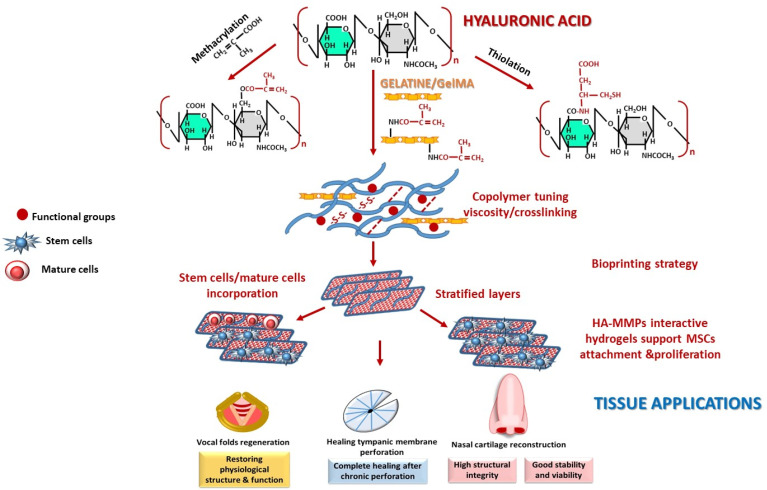
Structures and ENT applications of HA GAG where GlcA moiety is shown in green and GlcNAc is shown in gray (GAG = glycosaminoglycan; HA = hyaluronic acid; GlcA = glucuronic acid; GlcNAc = N-acetylated glucosamine; MMPs = matrix metalloproteinases).

Among GAGs, hyaluronic acid (HA) is widely used as a biomaterial in ENT. HA is constituted by repeating disaccharide composed of glucuronic acid (GlcA) and N-acetylated glucosamine (GlcNAc) joined by an alternating β(1-3) glycosidic bond. The glycosidic linkage between GlcNAc and the subsequent GlcA unit is of the β(1-4) type (Figure 2). The native HA molecule is non-sulfated and does not belong to any proteoglycan [48,49,50,51,52,53]. To enhance properties as a biomaterial, HA can undergo several modifications, including deacetylation of the -NHCOCH_3_ group, the covalent crosslinking of -OH and -COOH moieties, and the conjugation of free functional groups to generate esters, ethers, amides [49,54,55] and facilitating hydrogel formation [56,57]. Consequently, HA serves as a versatile scaffold in tissue engineering [57,58]. In ENT, HA-based hydrogels are employed in TM repair [34,40,59,60] and the reconstruction of vocal cords, trachea [40,59,61,62], and endoscopic sinus surgery [8]. For example, HA-derivative hydrogels applied for vocal folds regeneration in animal models demonstrate benefits in restoring physiological structure and function [63]. Another study highlights that a hydrogel composed of 1% HA-methacryloyl, 15% GelMA, and 24% ECM achieved complete healing of chronic TM perforation in rats one week after implantation [64].

**Figure 3:**
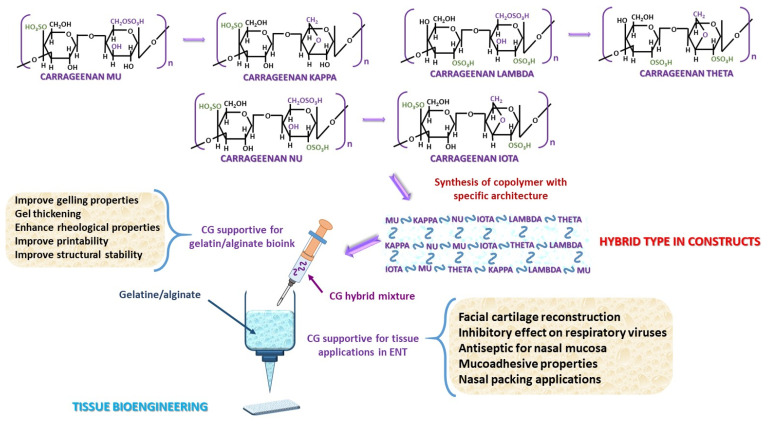
GAG-like configuration of primary CG types. The site of 3,6-anhydride bridge formation is highlighted in purple, while the sulfation sites of each Gal moiety are indicated in green (GAG = glycosaminoglycan; CG = carrageenan; Gal = galactose).

Carrageenan (CG) is a glycan with a GAG-like structure used as a biomaterial. CG is composed of sulfated galactose (Gal) units and exhibits greater structural similarity to GAGs than to typical homopolymers. Unlike GAGs, CG does not contain hexuronic acid (glucuronic or iduronic acid). Depending on the sulfation pattern, several types of CG have been identified; the most prominent are: Mu, Nu, Lambda, along with their 3,6-anhydride containing bridge derivatives Kappa, Iota, and Theta, respectively [65,66]. The precursor forms are considered to be non-bridge equivalents of the 3,6-anhydride variants (Figure 3). The 3,6-anhydride bridge promotes the formation of helical structures within the CG chain, which leads to the development of a gel network [67]. The transformation can be carried out either enzymatically in vivo or at alkaline pH in vitro [68]. Between monosaccharides, β(1-4) and α(1-3) glycosidic bonds are established alternatively [65,66,69]. In the natural environment, these CG structures exist as a hybrid type [70,71,72]. The most common Iota and Kappa forms are most commonly utilized for their gelling properties and stability [67,72], while CG Lambda is primarily used as a thickening agent [67]. Generally, CG hydrogels exhibit limited mechanical stability, and the resulting constructs often lack structural stability [33]. It has been observed that incorporation of CG into alginate hydrogels enhances their rheological properties, leading to improved structural stability and printability [33,73]. In ENT, Iota CG, containing antiseptic solutions applied to the nasal mucosa [74], demonstrates an inhibitory effect on rhinovirus, influenza A, and SARS-CoV-2 [74,75,76]. Scaffolds based on gelatin–CG can serve as biodegradable, biocompatible materials for nasal packing applications due to mucoadhesive properties [77]. Similarly, Kappa–CG polyvinyl alcohol hydrogels have been developed for facial cartilage reconstruction [78].

#### 2.2.2. Alginate

**Figure 4:**
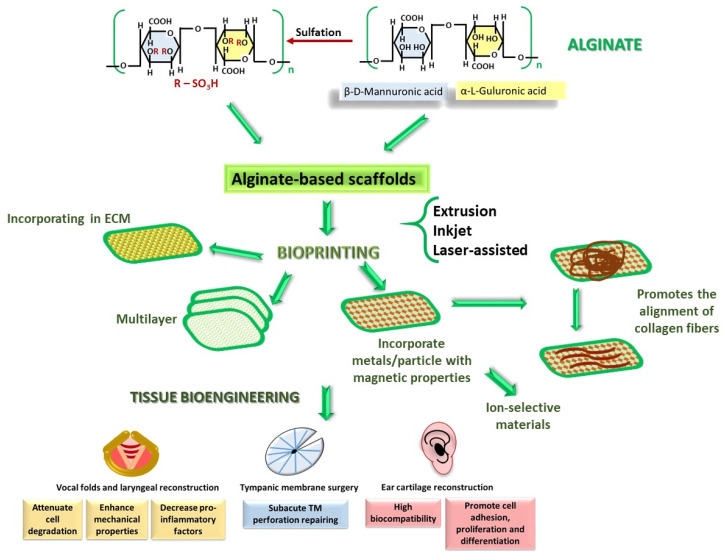
Alginate chemical structure and its applications in otolaryngology(blue—mannuronic acid moiety; yellow—guluronic acid moiety; ECM—extracellular matrix).

Alginate is composed of β-D-mannuronic acid and α-L-guluronic acid units linked by β(1-4) and α(1-4)glycosidic bonds, arranged either in alternative sequences or in domains containing homopolymer fragments (Figure 4) [79,80,81,82].

This structure can form hydrogels in the presence of divalent ions Ca^2+^, Zn^2+^, Cu^2+^, and Mg^2+^ [81,82]. The free -OH groups are susceptible to sulfation; the resulting alginate-sulfate composition supports cell proliferation and migration within the extracellular matrix (ECM) [83]. In general, alginate-based scaffolds and hydrogels have a wide range of applications both in regenerative medicine and as antimicrobial biomaterials. These materials find applications in various fields, including dermatology [84,85], oncology [84], and the repair of cartilage and bone tissues [85,86]. In ENT, alginate-based scaffolds have been applied in cartilage and bone tissue engineering [83]. Alginate-containing hydrogels incorporated into the ECM extracted from vocal fold mucosa enhance mechanical properties and attenuate cellular degradation. These hydrogels also lead to a decreased generation of pro-inflammatory factors and reduce angiogenesis in Sprague-Dawley rat models [87]. In a study involving canine models of various breeds, alginate-containing biomaterials were applied to a unilateral laryngeal injury, while the contralateral part servedas an internal control. Here, a significant improvementin the healing area treated with these biomaterials was observed compared to the control, as evidenced both microscopically and macroscopically [11]. Other authors have demonstrated the efficacy of alginate-based scaffolds in the repair of subacute TM perforations [40]. Thus, mixed scaffolds of polylactic acid with 3% sodium alginate or 3% chitosan proved superior to those containing only polylactic acid for 3D printing the TM [88].

#### 2.2.3. Gellan Gum (GG)

**Figure 5:**
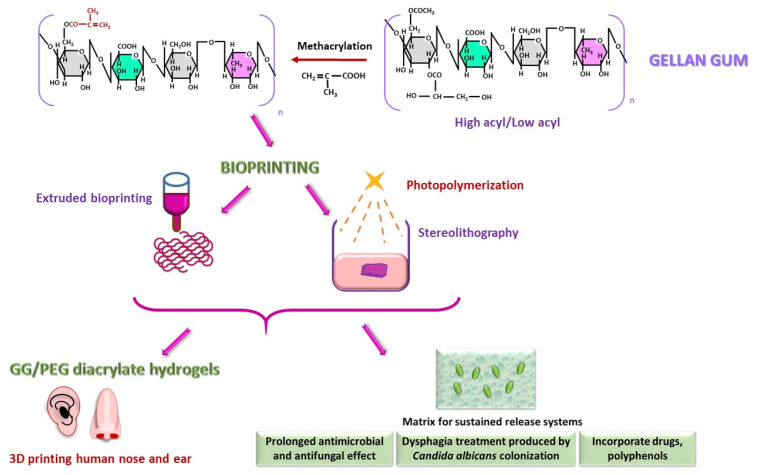
Chemical structure and schematic representation of gellan gum for ENT applications. Glucose and derivatives are illustrated in gray, the glucuronic acid residue is shown in green, and rhamnose is highlighted in pink/magenta (GG—gellan gum; PEG—polyethylene glycol).

GG is a heteropolymer consisting of a repeating tetrasaccharide unit composed of glucose (Glc), GlcA, Glc, rhamnose linked by β(1-4) or β(1-3) bonds. Glycerate or acetate residues may be esterified to the -OH groups of Glc in C-2 and C-6 positions (Figure 5), which significantly influence the polymer’s gelation properties. Depending on the abundance of these residues, GG is classified as high acyl or low acyl [89,90,91]. The stability of GG hydrogels can be enhanced by conjugating molecules to the free -COOH or -OH groups. In particular, functionalization with methacrylate allowed these polymers to undergo a photopolymerization process [92]. The metacrylation transforms GG into a compound highly relevant for tissue engineering and biomedical issues [93]. For instance, GG/PEG diacrylate-based hydrogels are suitable for 3D printing human ear and nose cartilage [94]. GG hydrogels can also serve as matrices for sustained-release systems; when applied locally to the mucosa, these systems provide prolonged antimicrobial and antifungal effects [89,95,96]. Furthermore, GG can incorporate various polyphenols such as caffeic acid phenethyl ester and ellagic acid, which show potential in treating dysphagia caused by *Candida albicans* colonization [96,97]. One hypothesis is the development of biofilms incorporating naturally derived bioactive compounds, given that certain food products are rich in phenolic content [98,99,100].

#### 2.2.4. Chitosan

**Figure 6:**
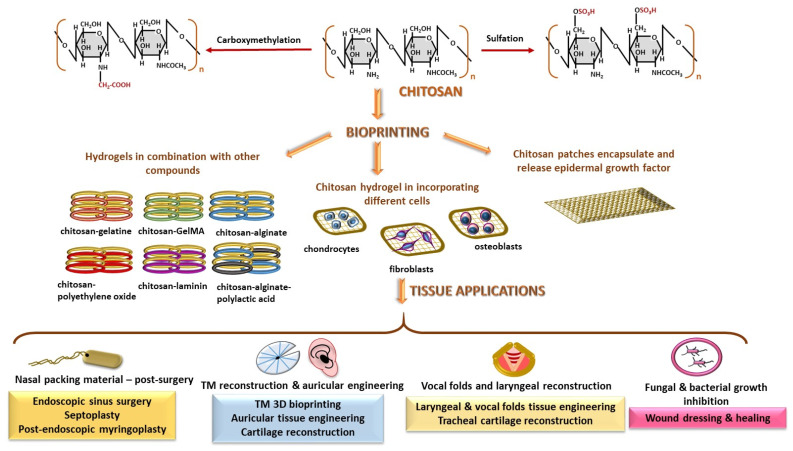
Importance and structural configuration of the chitosan repeating disaccharide unit with glucosamine derivatives highlighted in gray (TM—tympanic membrane; GelMA—gelatin-methacrylate).

Chitosan is a glycan formed by the polymerization of glucosamine and GlcNAc linked by β(1-4) glycosidic bonds (Figure 6), which are randomly distributed along the polysaccharide chain [101,102]. Chemical modifications are facilitated by the presence of free -OH and -NH_2_ groups, resulting in more robust and stable polymers [103,104]; these modifications include phosphorylation, sulfation, carboxymethylation, etc. [104,105,106]. Structurally modified chitosan hydrogels can serve as scaffolds in regenerative medicine and are widely applied in wound dressing and healing [103]. It has been observed that the incorporation of hydroxyapatite into chitosan hydrogels effectively stimulates the bone [106,107] and cartilage regeneration [107,108]. In addition, exposure of the protonated -NH_3_^+^ group and carboxymethylation inhibit the growth of several microbial species, including *S. aureus* [103,104,105], *E. coli* [103,105,109], *B. subtilis* [103], *Candida albicans* [105,109]. Recent research in otolaryngology is focused on novel cartilage tissue engineering strategies such as cultures of auricular chondrocytes on chitosan-based scaffolds [59], the reconstruction of tracheal cartilage using chitosan-embedded matrices [110], the application of chitosan patches for TM reconstruction [8,60], and post-surgery wound healing [45]. Chitosan is also used in combination with laminin in various experimental models of recurrent laryngeal nerve regeneration [111] and vocal fold tissue engineering applications [112]. Chitosan-based nasal packing materials are involved following endoscopic sinus surgery [113,114,115], septoplasty [116], while chitosan-ear packing materials are used post-endoscopic myringoplasty for treatment in chronic suppurative otitis media [117].

Polysaccharides generally exhibit a biocompatible structure susceptible to chemical modifications that enhance their functional properties. These glycan polymers are widely used as scaffolds, enabling encapsulation of pharmacologically active molecules or regenerative agents for reconstructive surgery applications. Furthermore, specific polysaccharide structures can prevent bacterial adhesion and colonization, thereby directly or indirectly improving the prognosis of patients receiving carbohydrate-related biomaterials.

### 2.3. Peptides and Proteins

Protein-based biomaterials represent a rapidly expanding field in reconstructive, regenerative medicine, and anti-aging treatments. Certain molecules serve as structural and/or functional foundations in different medical tissue bioengineering strategies in general medicine and in otolaryngology. Among these, collagen, gelatin, and silk proteins are the most utilized.

#### 2.3.1. Collagen

Collagen is a triple-helix protein and the most abundant component in ECM, accounting for approximately 30% of the body’s total protein mass [118,119]. Of the 28 types of collagen, types I, II, III, V, and XI are the most prevalent in the human body. This is composed of a repetitive tripeptide sequence of GXY type, where G = glycine, X = proline, and Y = hydroxyproline with left-hand winding [118,120,121,122]. As a biomaterial, natural collagen hydrogels exhibit hemostatic properties when applied during bleeding procedures [123]. Collagen is recognized for its ability to promote the healing of tympanic membrane perforations [124]. Since collagen types I, II, and III are intrinsic parts of the tympanic membrane, they represent an important material for tympanoplasty [34,40]. In an experimental animal model featuring acute and chronic TM perforations [125], collagen grafting was associated with superior recovery and regeneration of the TM, demonstrating high biocompatibility potentials. In another study [126], a cohort of 60 patients with tympanic perforation was monitored after the application of a collagen patch, yielding similar success rates as traditional methods. It is noteworthy that natural fat grafts, often obtained from lipoaspirates [127], serve as an important source of stem cells [127,128]. Collagen-based materials are used in laryngoplasty, as the vocal folds are inherently rich in type I and III collagen [129], while vocal fold scars accumulate an excessive content of collagen type I [60,61]. Tracheal tissue [20,60] and nasal cartilage [8] engineering procedures use collagen. A systematic review [130] noted that collagen types I and II are primary substrates used as scaffolds for nasal cartilage 3D bioprinting [130]. Although the majority of these studies highlight collagen’s capacity as a scaffold in reconstructive ENT treatments, certain patient-based factors can negatively affect the final clinical outcome, for instance, high alcohol consumption [131].

#### 2.3.2. Gelatin

Gelatin, a protein derived from collagen hydrolysis, represents a valuable biodegradable material in regenerative medicine. Unlike collagen, which has a triple-helix structure, gelatin consists of a single-stranded chain stable within a pH range of 5–9 [132,133]. The physical and mechanical properties of gelatin can be improved by chemical modifications. Among these, polymerization with meth acrylamides produces gelatin-methacrylate hydrogels (GelMA) with superior stability, used in tissue regeneration and repairing technology [33,86,134]. GelMA incorporates several polysaccharides, such as HA [135,136], chitosan [137,138], and alginate [139,140], to suit diverse biomedical applications. In otolaryngology, HA incorporated in GelMA has been used for nasal cartilage regeneration [36] and TM perforations healing [60], while gelatin–silk bioinks have been applied for printing auricular cartilage [15]. When gelatin and chitosan were utilized as packing agents in endoscopic myringoplasty, no significant difference in therapeutic outcomes was observed between the two groups [117]. The use of gelatin sponge for nasal deformity correction following epidermoid cyst excision resulted in excellent tissue adhesion, enhanced healing, and superior hemostatic properties [141].

#### 2.3.3. Silk Proteins

Silk proteins, especially silk fibroin (SF), consist of a heavy chain and a light chain linked by an S-S bridge. Within the heavy chain, a repeating dipeptide motif of Gly–X is observed, where X is predominantly; these sequences frequently terminate in dipeptide Gly–Ser or Gly–Tyr patterns [142,143]. In the crystalline state, the secondary structure is dominated by antiparallel β-sheets (silk II), which confer good stability in aqueous media. Amorphous forms consist of α-helix and random coil arrangements (silk I) [142,144]. The biodegradation of SF occurs via enzymatic hydrolysis and is dependent on the β-sheets content, which represents a more resistant structure. This degradation profile can be adjusted by techniques that promote the transition from silk II to silk I, a transformation produced in the presence of methanol, ethanol, KCl, or γ radiation [10,142]. This property allows the preparation of diverse SF-based scaffolds such as hydrogels, films, fibers, and sponges, as well as other biorientable materials [10,142,145]. SF-based materials can serve as scaffolds in otorhinolaryngology to facilitate cell proliferation in TM regeneration [34] or as components in absorbable drainage tubes for the evacuation of ear effusions in otitis media [146]. A 2:1 polycaprolactone/SF copolymer designed for TM implantation demonstrated the ability to fully restore the oscillatory properties compromised by perforation [147]. Despite the rapid healing of TM perforations, the complete degradation of SF-containing scaffolds post-implantation ranges from 6 months to one year [35].

Protein-based biomaterials are generally biocompatible and biodegradable scaffolds used in tissue regeneration. These materials represent a versatile source that can be manipulated by adding or removing functional groups or molecules to adapt them to specific individual clinical requirements.

Moreover, certain biomaterials are suitable for 4D bioprinting, as they can adapt to environmental conditions and respond to complex stimuli. This capability opens up opportunities in regenerative medicine, prosthetics, and beyond.

## 3. Cell Types in Tissue Regeneration

During the bioprinting process, the constructed scaffolds are populated with cells. In ENT applications, the selection of these cells is related to the target anatomical site, the specific tissue involved, the nature of the defect requiring regeneration or replacement, and the degree of maturity and differentiation. Based on these criteria, the cell sources are generallyclassified into stem cells and mature cells.

### 3.1. Stem Cells

Stem cells can be embryonic stem cells (ESCs), induced pluripotent stem cells (iPSCs), or mesenchymal stem cells (MSCs) [146].

ESCs can be cultured indefinitely in vitro, serving as valuable tools in directing differentiation into various specialized cell types [148], and areconsidered the first stage of pluripotency [149]. Their lineage commitment is conditioned upon the provision of an appropriate growth medium and the modulation of transcription factors [150]. ESCs used for 3D bioprinting can be derived from human blastoids, amniotic fluid, or primary yolk sac and can differentiate into any somatic cell [151,152]. Rather than merely serving as volume- filling agents, these cells are intended to create functional tissue architectures with vascular networks and complex neuronal connections. For example, 3D neural structures have beenconstructed from mouse ESCs in combination with brain-derived ECM background incorporated into Geltrex hydrogel (containing collagen, laminin, heparan sulfate, entactin). The authors demonstrated that the addition of brain-derived ECM in Geltrex hydrogel promotes the differentiation of mouse ESCs, whereas it does not have the same effect on neural stem cells [153]. In ENT, epithelial cells derived from ESCs show great promise in the reconstructive surgery of the vocal folds [111] and the inner ear [154]. Thus, for the treatment of hearing loss, human ESCs can be differentiated into inner ear hair cells [155]. Furthermore, the neuronal differentiation of otic neuronal progenitor-derived ESCs within nanofibrillar cellulose hydrogel facilitates their in vivo transplantation into the inner ear [156].

Similar to ECSs, iPSCs possessed the ability to differentiate into specific mature cells. They originate from specialized cells “rejuvenated” to the embryonic behavior, through induction by reprogramming factors such as octamer-binding transcription factor 4 (OCT4), sex-determining region Y-box2 (SOX2), Kruppel-like factor 4 (KLF4), and cellular-myelocytomatosis (c-MYC) [157,158,159]. These cell types remove the ethical controversies raised by the use of human embryonic cells; however, they carry a risk of malignant transformation during manipulation [159,160]. In regenerative medicine, iPSCs are used for their ability to differentiate into a wide range of tissues such as cartilage [158], blood vessels [159], skeletal muscle [161,162], glial cells [163], skin tissue [164], and develop complex organoids [165,166]. In ENT, Van der Valk et al. demonstrated the utility and potential of inner ear organoids (IEOs) for investigatingthe vestibular and cochlear diseases by generating a single-cell atlas of inner ear tissues [167]. Otic progenitor cells derived from iPSCs have proven value in modeling the impact of the ototoxic effect of drugs such as gentamicin and cisplatin on the inner ear [168]. Heparinized gelatin-derived hydrogels can induce the transformation of ESCs into iPSCs. In one study, norbornene-functionalized gelatin cross-linked with thiolated macromolecules such as polyethylene glycol (PEG) and HA, and functionalized with heparin induced evolution towards ectoderm, mesoderm, and endoderm [169]. For example, IEOs obtained by inducing iPSCs on different hydrogels, such as Matrigel (formed of laminin, collagen, entactin, and perlecan) or GelMA in combination with HA, showed significant therapeutic potential for patients with hearing loss [170]. In addition, the production of human otic neuronal organoids [171] and the mapping of developmental stages of IEOs [172] required Matrigel supplementation.

MSCs are multipotent cells that can be isolated from different tissues such as bone, cartilage, adipose, and neuroglial tissue. They are used in regenerative medicine due to their ability to self-renewal, their capacity to differentiate into organ-specific cell types within a proper environment, their immunomodulatory properties [173,174], and their tendency to migrate to injured tissue via chemoattraction [175]. MSCs have broad clinical applications in neurological diseases (multiple sclerosis, stroke, Alzheimer’s disease), cardiovascular diseases (heart failure, ischemic heart disease), respiratory diseases (chronic obstructive pulmonary disease, respiratory distress syndrome, bronchial dysplasia), endocrine diseases (diabetes mellitus due topancreatic dysfunction), skin burns, and wound healing [176]. Ethical concerns are diminished comparedwith those of using ESCs and, unlike iPSCs, the risk of malignant transformation is much lower [175]. In otology, MSC-derived extracellular vesicles (EVs) have been used to treat inner ear cells, protectingthem from the cytotoxic effect induced by chemotherapy. Perde-Schrepler et al. [177] demonstrated this effect using the HEI-OC1 cell line. Incubation of the HEI-OC1 cell line with MSC-derived EVs in different concentrations for 24 h produced an increase in cell viability upon cisplatin treatment, EV dose-dependent. Compared to the control group, the most intense effect was observed at an EV volume of 30 µL [177]. Other MSCsources, such as those derived from adipose tissue, bone marrow, and cartilage tissue, have a therapeutic potential in auricular cartilage regeneration [17]. Thus, adipose-derived MSCs obtained by liposuction are co-cultured with auricular chondrocytes in a 5:1 ratio, representing an effective choice for the de novo construction of auricular cartilage using type I collagen bioink. Following an eight-week culture period and subsequent implantation in immunodeficient Hsd:RH-Foxn1^rnu^ rats, the formation of cartilage tissue in vivo was visible in 2 months after implantation [178]. In laryngology, the regeneration of laryngeal cartilage and vocal foldsrepresents a challenge. A study conducted in nude rats demonstrated that involving MSCs derived from iPSCs resulted in laryngeal cartilage regeneration in 66.7% of the animals following total laryngectomy [179]. Similarly, another study [180] achieved laryngeal cartilage regeneration using human iPSCs -derived MSCs in immunodeficient X-SCID rats. A comparable outcome was obtained using adipose tissue-derived MSCs, facilitating laryngeal cartilage regeneration. Following the surgically induced laryngeal injury in Dutch rabbits, it was observed that the defect was repaired after 28 days, characterized by a reduction in fibrous tissue in the absence of laryngotracheal stenosis [181].

These stem celltypes hold potential in diverse applications. The selection of a specific stem cell type depends on the research objectives, requiring carefulevaluation of the advantages and disadvantages ofeach type, their availability, and interaction with the scaffold, as well as the dimensions of the target tissue(s)/tissue fragment. Asummary with these characteristics is provided in Table 1.

### 3.2. Mature Cells

Although mature cells derived from tissues such as bone and cartilage can produce ECM and can be used in ENT regenerative applications, their clinical use remainslimited by several constraints. In the case of bone tissue, current in vitro protocols involve co-culture of osteoblasts and osteoclast precursors to evaluate their behavior and the potential for applications in bone regeneration. The authors recommend the use of trabecular or cortical bone for the isolation of human cells [191]. If osteoblasts are incubated for 72 h on a peptide-functionalized chitosan support, they exhibit clear osteoblast adhesion followed by the generation of elongated shapes and subsequent proliferation and differentiation [192]. Cartilage repair strategies often involve the use of mature chondrocytes embedded within various hydrogel scaffolds such as collagen, gelatin, HA, PEG, fibrin, alginate, or agarose [193]. Alternatively, chondrocytes can be seeded in polyglycerol sebacate methacrylate polymeric high internal phase emulsion microspheres. This technique facilitates the production of a dense ECM, with the generation of a cartilage-like structure characterized by a high content of GAGs and type II collagen [194]. For instance, microtia-derived chondrocytes have been utilized to reconstitute mature cartilage using a tri-layered scaffold consisting of a polycaprolactone core coated with polyglycolic acid and polylactic acid outer layer [195]. Additionally, autologous nasal cartilage has been applied to the repair of nasal septal perforation [196]. Even though osteoblasts, osteoclasts, and chondrocytes have a significant potential for regenerating tracheal, auricular, and nasal defects, their applications are hindered by limited lifespan and the risk of dedifferentiation, which reducestheir capacity to generate ECM [15].

### 3.3. Synthetic (Artificial) Cells (SCs)

SCs are laboratory-constructed vesicles/particles designed to mimic the behavior of living cells or beyond their natural capabilities. For instance, SCs can process unnatural amino acids or toxic molecules that aretypically lethal tonativecells; alternatively, they can execute metabolic pathways that donot naturally occurin human tissues [197,198]. The introduction of a genetic code into SCs enables the autonomic protein synthesis. An example is the production of recombinant basic fibroblast growth factor, where a single cell can generate 9 × 10^6^ protein copies. The integration of these SCs into a construct containing endothelial cells, Matrigel, and type IV collagen has been shown to promote angiogenesis in BALB/c mice [199]. Furthermore, SCs hold potential for the synthesis of artificial ECM [200], the modulation of oncogenes in cancer therapy [201], or the biosynthesis of deficient enzymes [168]. RegardingENT applications, these materials show promise in vocal fold regeneration. Specifically, they can be utilized as polymeric alginate-polylysine or alginate-chitosan microspheres containing encapsulated fibroblasts [202]. Additionally, polymethylmethacrylate microspheres suspended in collagen have been used to stimulate fibroblasts in patients with unilateral vocal fold paralysis [203].

SCs provide new opportunities and open up avenues for finding solutions that were previously unattainable using traditional living biological systems. However, public acceptance and ethical concerns remain two important aspects of technology SCs. A study encompassing 13 European countries highlights a notable degree of acceptance for these agents among the general population [204]. The use of SCs must be governed by ethical principles to ensure that research and clinical implementation occur in a safe and regulated environment. Thus, several critical factors such as the risk/benefit ratio, safety and controllability, environmental protection, avoiding intentional or unintentional releases, justice principles, fair accessibility, and moral principles must be taken into account [204,205,206].

## 4. Bio-Printing and Engineering

Bioprinting and bioengineering have revolutionized the therapeutic approach across medicine and, in particular, in ENT. Biomaterial inks serve as the fundamental building blocks of future tissues. These materials are selected according to local functional and structural requirements as well as their mechanical resistance and biodegradability. Currently, technologies facilitate not only the construction of 2D tissue blocks but also the transition toward 3D and 4D bioprinting.

### 4.1. Three-Dimensional Bioprinting

Starting from a traditional 2D methodology, current tissue engineering involves 3D bioprinting to revolutionize regenerative and implant medicine [207,208]. This technology is based on biocompatible polymers described above as primary structure components forming scaffolds that are populated with different types of cells [208,209,210]. This ability to build 3D structures using living biological materials generally encompasses four main technologies: inkjet, laser-assisted, and extruded bioprinting, as well as stereolithography [209,210,211].

**Figure 7:**
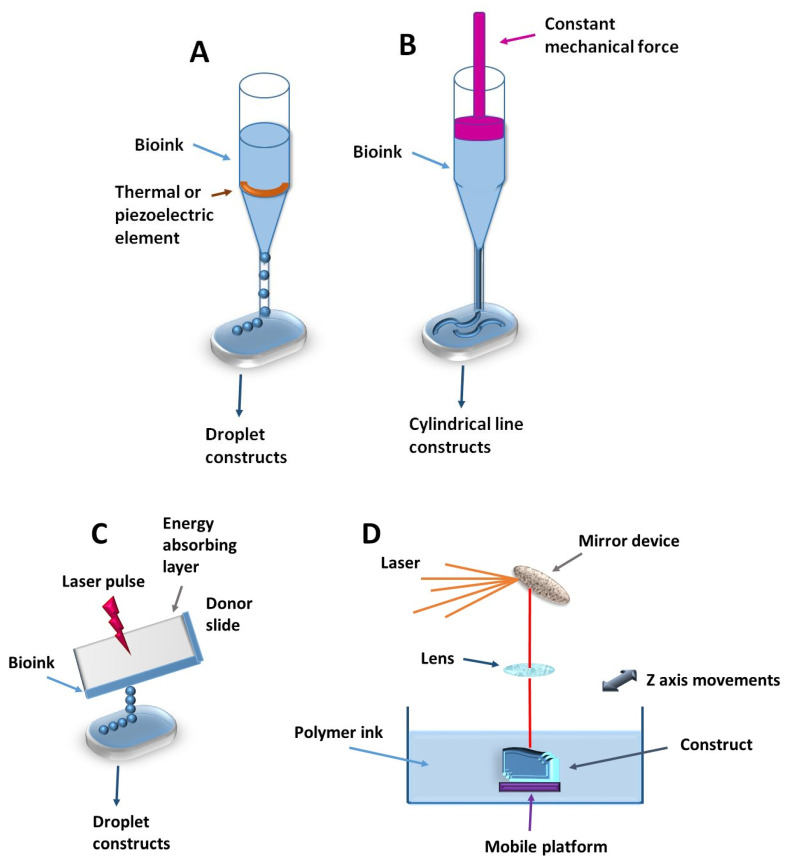
Schematic representation of the primary 3D bioprinting technologies: Inkjet (**A**); Extruded-based (**B**); laser-assisted (**C**) and stereolithography (**D**).

Inkjet bioprinting involves the deposition of cells in the form of discrete droplets driven by thermal or piezoelectric actuators and directly operated by a computer controller [208,209,210] (Figure 7A). This technique demonstrates high resolution, high-throughput capabilities, and excellent reproducibility, and its cell viability exceeds 90% [207]. A major challenge isthe material selection, which must be dispersed in a liquid state and maintain a specific viscosity [207,212,213]. Hydrogels represent an attractive choice as bioinks due to several advantages: they provide a protective environment for cell membranes against direct stress encountered during the bioprinting process. This stress could produce membrane rupture and irreversible injuries to cellular components and structures [214]. Another significant advantage is their cellular compatibility and the high water content that facilitates diffusion of nutrients, metabolic end-products, and molecules involved in cell signaling [212,214,215].

Extruded bioprinting is a variant of inkjet bioprinting in which a constant driving force is applied, resulting in a continuous cylindrical line product rather than discrete droplets [207,210] (Figure 7B). This method allows various materials of different viscosities, making it suitable for large-scale tissue engineering [207,216]. However, the cells are under additional stress, which can compromise their viability [208]. The limited spatial resolution renders this technique less suitable for fine structural details [216]. The materials used are calcium alginate hydrogels, GG, agarose, collagen gelatin, and GelMA [86].

Laser-assisted bioprinting uses high-pressure laser pulses to propel bioinks onto the printable surface (Figure 7C). The process is initiated from a donor slide covered by an energy-absorbing layer [210] upon which the bioink is applied. Because there is no physical contact between the bioink and the dispenser, the cells support a minimal stress, which results in increased cell viability of 90% or greater [207,216]. This method has limited practical applications and remains primarily in the experimental phase, due to the high cost and technical difficulties of maintaining droplet uniformity [216,217].

Stereolithography is one of the most precise bioengineering techniques utilizing a UV laser to photopolymerize and solidify the polymer structure in a layer-by-layer fashion [218] (Figure 7D). While this method offers high spatial resolution, is precise, and fast, it carries the risk of DNA and cell damage under UV exposure. This limitation can be mitigated by incorporating monomers containing alkene-type structures or thiol groups that can undergo spontaneous cross-linking on 266nm exposure [207]. The most commonly used materials as scaffolds are hydrogels containing polyD,L-lactide, polypropylene fumarate, polyethylene glycol diacrylate (PEGDA), and GelMA [219,220].

The application of 3D bioprinting methods is generally customized based on the specific pathology, the target anatomic structure, and the biomaterial used. Table 2 summarizes the advantages and disadvantages of each method. Bioethical considerations must not be overlooked, which, in the context of the transition from 3D to 4D, may raise debate issues.

### 4.2. Four-Dimensional Bioprinting

The transition from 3D to 4D bioprinting represents an evolution from relatively rigid constructs to smart materials capable of adapting and evolving under controlled conditions. As a next-generation complex process, 4D bioprinting is increasingly being explored for applications in regenerative medicine, implants, and advanced medical devices. In this way, the resulting 3D construct can integrate with the surrounding tissue and mimic its function, establishing true microphysiological systems [225]. This process is achieved through dynamic shape modification and functional transformations [226]. In this sense, printing materials must be engineered to respond to the complex conditions of the internal physiological environment. This triggers physical stimuli (temperature, humidity, magnetic field, electric field, UV and IR radiation) as well as chemical stimuli (ions, ionic liquids, pH changes, hydrogels, enzymes) [36].

Regarding physical stimuli, thermoresponsive agents must adapt to the temperature of the body’s internal environment. For instance, chitosan-based hydrogel can undergo sol–gel transition at body temperature [227]. Other thermoresponsive polymers with significant utility in biomedical applications are: poly-N-isopropylacrylamide (PNIPAM) or polyethylene oxide/polypropylene oxide block copolymer [228]. Humidity-responsive materials can change their size and shape depending on the water content at the application site. Hydrogels containing alginate and HA can be used in stratified layers and combinations to mimic the multilayered structure of various tissues, particularly cartilage [229]. Other hydrogels suitable for this application are PEG [230] and cellulose [229]. Light/UV-responsive materials can undergo photodegradation, suffering specific shape changes [230]. This property can be used for blood vessel-like networks inside hydrogels, creating microchannels that mimic native vascular structures [231]. Electric/magnetic field-responsive materials can undergo significant conformational changes in response to electrical or magnetic stimuli. Magnetic response materials incorporated metals or particles with magnetic properties within their polymer matrices [230,231]. Examples include alginate hydrogels used in gastric cancer research for mechanical pressure stimulation [227], iron oxide particles embedded in PNIPAM, polyvinyl alcohol, polyacrylamide, and alginate to facilitate the alignment of collagen fibers [228]. The most prominent polymers that respond to electrical stimulation are polythiophene, polyaniline, polypyrrole, and GelMA [229], which exhibit dynamic adaptability to cardiac electrophysiology [232] and enhance ECM plasticity [233].

Regarding chemical stimuli, pH variations mediated by pKa-dependent protonation-deprotonation phenomena can modify the bioinks. By modifying the ionization state of biopolymers, thesepH changes modify the solubility of some structures and influence the swelling of the hydrogel [234]. They can improve angiogenesis, migration, and proliferation of macrophages as well as influence fibrosis [235]. Among pH-sensitive polymers, polyacrylic acid, polysulfonic acid, poly-2-aminoethyl methacrylate, poly-N,N′-dimethyl aminoethyl methacrylate, polyethylene imines, and chitosan-polyethylene oxide have been investigated [228,234]. Divalent ions such as Zn^2+^, Ca^2+^ can produce reversible or irreversible structural changes in bioprinting constructs. For example, the Zn-imidazole interaction is reversible; the addition of a chelating agent can remove metal ions and transform the original configuration into an irreversible form [231]. Alginate-based hydrogels are suitable for developing ion-sensitive materials [228]. Magnesium alloys can be integrated with 4D bioprinting materials to promote bone healing, particularly in patients with osteoporosis [235]. Enzymes can mediate interaction between biopolymers and microenvironment structures, facilitating integration of these materials into native tissues [231]. Matrix metalloproteinases (MMPs) allow integration of PEG in host tissue [236]. HA-MMPs interactive hydrogels support MSCs attachment and proliferation by modulating the extracellular matrix properties [230]. Their increased specificity makes these systems adaptable for drug delivery [237].

Other stimuli include those that promote bioimplant degradation. Degradation is a natural process undergone by biocompatible scaffolds. However, bioprintable scaffolds must meet the following criteria: (i) maintain mechanical stability until tissue is complete; (ii) facilitate new tissue formation through their intrinsic properties or the sustained-release of bioactive agents; and (iii) ensure that degradation by-products are non-toxic and easily eliminated, preventing accumulation [238]. Degradation can be influenced by chronic mechanical stress exerted on the bioimplant as well as by local enzymatic activity, non-enzymatic hydrolysis, and metabolites or reactive oxygen species (ROS) that can oxidize the material [238,239]. This process is further intensified by chronic inflammation, which can compromise the bioimplant. Persistent cellular activation, the release of proinflammatory cytokines, and the generation of ROS can significantly alter the scaffold’s properties. Several strategies involve the incorporation of anti-inflammatory agents—such as dexamethasone, acetylsalicylic acid, or ibuprofen—or specific mediators like IFN-γ. These substances polarize macrophages and inhibit proinflammatory mediators, thereby supporting osteogenesis and cartilage repair [240,241]. Macrophage polarization can also be modulated by incorporating ions (such as Mg^2+^, Zn^2+^, Cu^2+^) into the scaffold, which are subsequently released into the microenvironment [240]. Furthermore, surface modification can modulate macrophage polarization by altering substrate stiffness. To this end, PEG substrates with varying degrees of stiffness or polyacrylamide gels with collagen coatings are commonly employed [242]. In contrast to destructive chronic inflammation, acute inflammation is an essential component of the post-implantation healing process. Immediately following implantation, a provisional matrix is formed that functions as a temporary barrier through protein absorption. This matrix is rapidly enriched with growth factors and cytokines, which subsequently trigger neutrophil recruitment [242].

The selection of stimuli-responsive 4D agents is driven by the requirements of personalized medicine and tailored to address contemporary challenges. The interactions between biomaterials and the cellular environment are fundamental to the future of functional tissues/organ fragments and organ replacement. The development of biomimetic systems may achieve a shift from implant to transplant, potentially overcoming the critical shortage of organ donors for patients with end-stage organ failure.

### 4.3. Three-Dimensional/Four-Dimensional Bioprinting Applications in Otolaryngology

In otolaryngology, organ reconstruction involves multiple tissues, including bone, cartilage, skin, and epithelial tissue, and local vasculature. Consequently, an implant may consist of a single tissue type or a multicomponent construct. The latter requires not only advanced technical resources but also refined surgical expertise and a multidisciplinary approach.

#### 4.3.1. Nasal Reconstruction

The reconstruction of nasal and septal cartilage, as well as rhinoplasty procedures, are key focuses of 3D bioprinting research. To construct cartilage, adipose-derived stem cells or other MSCs are combined with several types of hydrogel bioinks; these combinations ensure stability, shape fidelity, and integration into the surrounding native tissues [243,244,245,246,247]. MSCs have been co-cultured with chondrocytes and integrated into various scaffolds [244,245,246]. The most suitable hydrogel formulations are those based on HA, SF, polycaprolactone, collagen, polyacrylic acid, PEG, PEG dimethacrylate, and GelMA [244,245]. In a study [248], human Naso septal chondrocytes (hNCs) were isolated from healthy volunteers and integrated intoa type I collagen matrix. This bioink was processed by the pneumatic-microextrusion method. After 6 weeks of culture period, the resulting nasal cartilage construct demonstrated high structural integrity and viability in vitro [248]. Similar results were reported by Lan X et al. [209] utilizing the same bioprinting method with a type II collagen scaffold. The same research group extended their investigations by evaluating several types of scaffold compositions for hNCs: methacrylated collagen(I), methacrylated collagen + thiolated HA (II), and methacrylated collagen + thiolated HA + PEG diacrylate (III). The authors found that all three variants support ECM formation without inducing excessive protein synthesis. The formulations containing thiolated HA exhibit an increased water-containing capacity due to the abundance of -COOH groups, which contribute to hydrogen bonding with water molecules. Furthermore, these variants were characterized by cell-mediated contraction and reduced condensation. The authors conclude that while variant III is the most promising candidate, the proportion of its three components should be optimized, as the 1:1 ratio in this study may not be ideal [249]. A critical consideration for further research is the functional integrity of the nasal epithelium. Deniz Derman I et al. [250] developed functional nasal epithelial models using droplet-based bioprinting with human nasal epithelial cells (hNECs). hNECs isolated from healthy volunteers were suspended in a type IV collagen hydrogel and bioprinted at a density of 1.1 × 10^5^ cells/insert. Although the viability was reduced to 1/2-1/3 compared to manual seeding, five distinct cell populations were identified, including goblet cells and multiciliated cells, with successful mimicking of native nasal epithelial tissue [250]. Nasal organoids are currently under intensive investigation for their potential to regenerate damaged olfactory epithelium in chronic rhinosinusitis as well as in neurodegenerative disorders such as Alzheimer’s and Parkinson’s disease. While iPSCs are a primary cell source for these organoids, tissue-specific stem cells can also be utilized [251]. Several mechanisms have been identified that influence olfactory epithelium regeneration. Specifically, the overexpression of chitinase-like 4 plays a critical role in this process, as demonstrated in organoids derived from mice of various ages [252]. Furthermore, it was observed that EGR1 overexpression in olfactory organoids promotes neuronal proliferation and differentiation [253]. These organoids represent a promising frontier in reconstructive medicine and ideal candidates for bioprinting.

#### 4.3.2. Ear Reconstruction

3D reconstruction of the auricular cartilage is essential in treating auricular atresia or other congenital or acquired deformities. This emerging therapeutic approach of reconstructive surgery increasingly incorporates 3D/4D bioprinting. To achieve this, various natural and synthetic non-resorbable biopolymers such as polyglycolic acid, polylactide, PEG, polylactic-co-glycolic acid, polycaprolactone, alginate, and collagen are utilized. These scaffolds are populated with co-cultures of chondrocytes and adipose-derived stem cells to promote tissue formation [36,146,254]. Researchers are seeking an ideal scaffold for auricular bioprinting; recent findings identified a 5:5 mixture of acellular cartilage matrix and sodium alginate as the optimal formulation. This specific ratio demonstrates superior performance in terms of biocompatibility, adhesion, proliferation, and cellular differentiation, as well as lack of cytotoxicity [255]. A bionic auricular scaffold was designed to mimic the native biomechanical properties of auricular cartilage. The construct containing polyvinyl alcohol and gelatin embedded in SiO_2_ was designed to support cell adhesion and proliferation [256]. Following the creation of a surgical defect in the ear of New Zealand white rabbits, the material was implanted to repair the site. Twelve weeks post-implantation, promotion of chondrocyte growth and cartilage regeneration were observed [256]. A similar study, conducted on the same type of rabbits, useda hyaluronan transglutaminase-based bioink encapsulated with autologous auricular chondrocytes to evaluate the material functionality and tissue compatibility [257]. Other investigators employed GelMA and methacrylated HA scaffold for auricular cartilage bioprinting, integrating co-cultures of human septal chondrocytes and human bone marrow-derived MSCs [258]. Several experiments have investigated the long-term viability and regeneration of the bioprinted auricles following subcutaneous implantation in mice with observation periods extended up to 24 weeks [259,260,261,262]. Expanding onthis work, Xie X et al. bioprinted auricular cartilage using microtia-derived tissue harvested from children [263]. Microtia-derived chondrocytes were expanded in culture, embedded within acellular cartilage matrix, and subsequently integrated into GelMA hydrogel. The resulting cell-laden bioink was bioprinted using digital light processing bioprinting technology. Following subcutaneous implantation in nude mice for twelve weeks, the neocartilage demonstrates an increase in the number of chondrocytes and collagen fiber deposition [263]. For the treatment of tympanic membrane (TM) perforation, 3D bioprinting technology is able to restore acoustic and mechanical properties. Typical scaffolds for this application contain GelMA, polycaprolactone, and polylactic-co-glycolic acid [264,265]. Alternative therapeutic approaches use chitosan patches to release epidermal growth factor [8] or containing MSCs for TM reconstruction [40]. Significant challenges also remain in the regenerative medicine of the inner ear. For instance, one experiment involved the development of the organ of Corti organoids within a GelMA and stearyl acrylate hydrogel [266] because these hair cells lack regenerative capacity [61]. Several GelMA–HA–Arg–Gly–Asp-based hydrogels have been developed to support the integration of cochlear organoids and provide ECM biochemical guidance for sensory epithelium formation [267]. Other hydrogels optimized for cochlear organoids integration include those based on gelatin-hydroxyphenyl propionic acid and Matrigel, which provide a microenvironment that facilitates adequate vascularization for organoid maturation within the inner ear [268]. Several studies utilized cochlear organoids to elucidate the mechanisms underlying hair cell regeneration. One such mechanism involves the activation of glycolysis through the overexpression of pyruvate kinase M2, as demonstrated in a transgenic mouse model [269]. It has also been confirmed that functional synapses can be established between hair cells within cochlear organoids [270]. In general, this segment of the ear remains less explored.

#### 4.3.3. Tracheal Reconstruction

Tracheal reconstruction utilizes scaffolds composed of collagen, HA, polycaprolactone, polyglycolic acid, polyurethane, polylactic-co-glycolic acid, which promote cellular attachment and proliferation [271,272,273,274,275]. These constructs were seeded with chondrocytes and MSCs, which were initially expanded in culture to form multicellular spheroids. These spheroids were precisely positioned within the scaffold using a fully automated robotic system [273]. Using fused deposition modeling technology, apolycaprolactone artificial trachea was fabricated 2.8 times faster than conventional methods while maintaining a rotation angle of 254° [276]. Another study reported the fabrication of a tracheal construct by extruded bioprinting employing polycaprolactone/3% atelocollagen bioink encapsulated with human nasal chondrocytes and human nasal turbinate-derived stem cells. A significant contraction in volume alongside the cessation of proliferation after seven days was observed exclusively at a density of 1 × 10^7^ cells/mL; notably, these effects were absent at lower concentrations. The authors consider that the optimal cell density for this application is 5 × 10^6^ cells/mL. The O-shape trachea was implanted subcutaneously in BALB/c nude mice to assess its chondrogenic potential, which was successfully validated [277]. Other authors focused on the reconstruction of C-shaped trachea [278,279,280]. Initially, C-shaped rings fabricated by extrusion bioprinting and connected by vascularized fibrous tissue were preimplanted subcutaneously in nude mice. The scaffold was formulated from a hydrogel mixture of GelMA, methacryloyl chondroitin sulfate, and methacryloyl elastin. The matrix was populated with chondrocytes and fibroblasts harvested from the auricular cartilage of New Zealand white rabbits. Following the preimplantation period, the constructs were implanted into the rabbits by end-to-end anastomosis. Eight weeks post-surgery, the tracheal construct exhibits a continuous, tubular, and functional structure successfully integrated in the native trachea [278]. Other similar studies present similar outcomes using the same strategy with minor modifications in C-ring composition. These formulations include HA methacrylate, 8-arm-PEG-succinic acid ester, and methacryloyl modified derm acellular matrix [279] as well as glucuromannan-peptide copolymer [280]. Other researchers studied the ferret model for laryngotracheal reconstruction involving ferret MSCs [281].

Promising advancements in 3D/4D bioprinting within the field of ENT open up perspectives for translational research, moving from in vitro and in vivo animal-based studies to exploring the behavior of these bioimplants in humans. The challenges remain complex, encompassing long-term biocompatibility, functional vascularization, and sustained functionality of the bioprosthesis.

## 5. Medical Applications in ENT Regenerative Medicine: Discussions and Future Perspectives

Innovations in reconstructive surgery have attempted to restore function and esthetic appearance to the damaged anatomical area. Over time, technical barriers have been overcome. Around the year 2000, nasal and auricular replantation with and without venous anastomoses, as well as tissue allotransplantation, were performed using microsurgical techniques [282]. In general, some techniques utilize autologous tissue transplantation while others employmetal prostheses or those made of other biocompatible materials. In the last decade, most of these procedures have integrated computer-aided design and computer-aided manufacturing [283]. Regarding autologous transplantation, several successful cases have been reported. For instance, in the case of an 81-year-old woman who underwent excision of the right lateral nasal wall due to basal cell carcinoma, the required cartilage was harvested from the right ear while the skin graft was obtained from a paramedian forehead flap [284]. Other compelling cases have been described regarding the three-layer reconstruction of full-thickness nasal alar defect resulting from the excision of basal cell carcinoma lesions [285] as well as for nasal reconstruction following traumatic amputation [286]. Titanium prostheses have been used as alternatives for extensive reconstructions. For example, 3D-printed titanium implants were utilized in the case of an 11-year-old boy who presented to the emergency room with a nasal avulsion and extensive laceration caused by a dog bite [287]. Additional reconstructive procedures involving titanium mesh implants have been performed on young patients with extensive bone loss, including a 19-year-old man with severe gunshot wounds and an 18-year-old woman with war-related traumatic injuries to the left side of her face [288]. Other synthetic materials, such as porous polyethylene or silicone, have also been successfully utilized. A porous polyethylene implant was placed in a 78-year-old patient who had lost a significant portion of his left auricle due to recurrent cutaneous basal carcinoma [289]. Auricular reconstruction using porous high-density polyethylene combined with a simultaneous implantation of a vibrating sound bridge and intraoperative audiological monitoring was successfully performed in a young patient with Grade III microtia and complete external auditory canal atresia [290]. A silicone prosthesis was utilized for a patient treated for mucormycosis, following a failed autologous bone graft that had undergone resorption [291]. Complications following the use of these materials and procedures have been relatively common. These include delayed events, such as the spontaneous fracture of a silicone implant in a 53-year-old woman one year after rhinoplasty [292]. Other drawbacks of silicone implants include capsular contracture, extrusion, and infection [293]. The use of flaps in auricular reconstruction has been associated with a complication rate of less than 10%, including postoperative necrosis, infection, and scar hyperplasia [294]. The limitations of these reconstructive methods—related to the laborious surgical techniques and postoperative complications—have led to the search for other alternative approaches, notably bioprinting.

The clinical applicability of 3D bioprinting in tissue bioengineering has been reported in several clinical cases. For instance, auricle reconstruction was performed in a pediatric patient with microtia involving autologous harvesting of a cartilage fragment from the first free-floating rib according to the Brent technique. This graft was subsequently implanted subcutaneously, preserving the local vascular plexuses to avoid necrosis. In the next stage, the neoauricle was reimplanted in the appropriate anatomical location [295]. The principal challenges associated with this technique include preventing pleural injury during costal cartilage tissue harvesting, hematoma, surgical incision infections, necrosis, and chest wall deformation. Long-term concerns involve the neocartilage growth commensurate with the child’s natural development. According to the authors’ experience, 48% of the pediatric patients exhibit growth comparable to the opposite ear, while 42% showed growth exceeding by afew mm the opposite ear, and 10% evidenced a slower growth [295]. An analog technique was applied to a 58-year-old man who had suffered a complete traumatic amputation of the ear. Although the patient was discharged with complete reconstruction, the authors provided no data regarding long-term postoperative evolution [296]. Another interesting case involved a 64-year-old man requiring subtotal nasal reconstruction following the resection of recurrent basal cell carcinoma. A porous polyethylene scaffold was initially prevascularized by implantation in the patient’s right volar forearm for two months. Subsequently, the construct was transferred to the facial defect, where arterial and venous anastomoses were performed to ensure local vascularization. The patient had a favorable outcome over 22 months of follow-up [297]. Similarly, a 66-year-old man who lost his nose under similar circumstances to the previous case was reported. The nasal reconstruction utilized autologous costal cartilage covered with a forehead skin flap. The evolution was favorable in one year, followed by a post-procedure [298]. In another case, stereolithography was employed to fabricate a polyether ketone scaffold for a 1-year-old child with arhinia. The reconstruction was performed in two stages, over a two-year period, and was clinically successful and well tolerated [299]. However, data regarding long-term outcomes and details related to this patient are missing.

Despite the high theoretical potential of these approaches, the clinical translationof tissue engineering faces several practical hurdles, including:

1. Long-term viability of the bioimplant. While bioprinted tissue has generally demonstrated good viability in clinical settings, the current literature lacks reports of procedural failures or cases with graft necrosis resulting from viability loss. Consequently, the absence of negative data precludes a truly objective assessment of the long-term success rates of these neotransplants.
2. Resistance to environmental factors (the extreme cold and heat in winter and summer) and minor trauma is a critical consideration, as the nose and auricles are anatomically prominent and frequently exposed to these stressors. These aspects were not quantitatively assessed in either successful clinical cases or animal models. Future research should prioritize evaluating the durability of bioprinted constructs, initially under controlled experimental conditions and subsequently in patients exposed to real-world environmental challenges.
3. Age-related modeling and senescence of bioprinted tissues. The long-term behavior of neotissues during the natural biological aging process remains unknown. Due to the novelty of these procedures, sufficient longitudinal data are not yet available to draw definitive conclusions. Further research in this regard is essential to ensure their lifelong performance in pediatric and adult patients.
4. Interaction with systemic pathologies and pharmacological treatments has not been reported in the literature. It is crucial to investigate how bioprinted neotissues respond to acute or chronic diseases compared to native tissue. Of particular interest are pathologies characterized by impaired perfusion accompanied by vasoconstriction and reduced local blood flow, as well as the effects of chronic hyperglycemia in diabetic patients. In these scenarios, a dual challenge arises ensuring the survival of native microvascularization while protecting the biomaterial from a potentially biochemically hostile microenvironment. In a specific pathological context, 3D bioprinted scaffolds can be tailored to better respond to these altered physiological conditions. Recent studies offer valuable insights into achieving this adaptation.
  − In diabetes mellitus (DM), the microenvironment characterized by hyperglycemia, inflammation, and mitochondrial dysfunction leads to the accumulation of ROS and advanced glycated end products (AGEs) [300,301]. Under these conditions, it is well established that, in addition to impairing microcirculation and promoting chronic inflammation, collagen glycation and the subsequent generation of AGEs are constant features in the progression of DM. This effect is also evident in collagen-based materials [302]. In patients with DM, several strategies exist for the management of 3D bioimplants. The use of decellularized ECM-based materials could prove effective under these altered conditions. Specifically, it is well established that chronic wounds are more alkaline than healthy skin, owing to bacterial contamination and an altered ECM. For instance, the development of pH-responsive hydrogels allows for the target release of vascular endothelial growth factor at alkaline pH (approximately 7.4) while inhibiting release at lower levels (5 or 6) [303]. One such 3D scaffold is composed of polyorganophosphazene polymers functionalized with antioxidant inclusions and fibroblasts. These scaffolds demonstrate enhanced cell viability and facilitate tissue regeneration in diabetic mouse models [304]. Recently, 3D GelMA-based scaffolds have been described that incorporate strontium-containing bioactive glass particles. These scaffolds are designed to remodel the diabetic tissue microenvironment, thereby enhancing angiogenesis, osteogenesis, and anti-inflammatory activity [305,306]. Certain hydrogels, such as those functionalized with glucose oxidase, have been designed to mitigate local hyperglycemia by catalyzing the conversion of glucose into hydrogen peroxide and glucuronic acid. This process subsequently lowers the local pH and exerts antimicrobial effects [307].
  − Hypoperfusion creates a chemically and metabolically hostile microenvironment. This leads to the accumulation of acidic metabolites, which accelerate the enzymatic degradation of polymers such as HA, alginate, and chitosan. Furthermore, the resulting reduction in local pH creates an environment that favors bacterial colonization [308]. In addition, the accumulation of acidic degradation products triggers the autocatalytic degradation of polylactic acid-based copolymers [239]. The reduction in local blood flow can induce ischemic conditions within the implanted biomaterial, leading to mechanical failure and the collapse of the scaffold [309].

A further area of clinical investigation would be related to the effects of medication and drug interactions on bioprinted neofragment, for example, the effects of ototoxic agents on bioprinted products versus native tissue or the hemodynamic response to local vasoconstrictors.

The considerations currently lack longitudinal validation to certify the lifelong efficacy of the structural transformations produced throughout the patients’ life journey. In the short term, there is a measurable improvement in the anatomical structure, physiological functionality, and esthetic outcome. However, the long-term effects resulting from the natural process of senescence and the gradual degradation of biomaterials remain unknown, representing a critical frontier for future investigations.

## 6. Conclusions

Tissue engineering biomaterials represent a transformative technology in regenerative medicine in general, with particularly high potential in otolaryngology. By using different biomimetic scaffolds that promote cell adhesion, proliferation, and differentiation, 3D/4D bioprinting ispoised to become the next therapeutic paradigm for advanced implant and transplant technologies. Although in vitro and in vivo animal models in ENT yielded promising results, the transition to human clinical applications presents unique challenges. These hurdles create diverse research opportunities; indeed, each section and subsection explored in this review identifies a potential avenue for advancing therapeutic interventions in ENT pathology.

## Figures and Tables

**Table 1:** The potential of stem cells in 3D/4D bioprinting applications.

Stem Cells	Applications	Advantages	Limitations	Cell-Scaffold Interactions
ESCs	Enable reconstructive surgery of the vocal folds using epithelial cells derived from ESCs [111]; Support reconstructive surgery of the inner ear [154]; Treat hearing loss through the differentiation of ESCs into inner ear hair cells [155]; Allow for the in vivo transplantation of ESCs-derived otic neuronal progenitors within cellulose hydrogels [156].	Possess the capacity to differentiate into any somatic cell [151,152]; Functionally create architecture with vascular network and complex neuronal connections [153]; Express pluripotent factors such as OCT4, SOX2 [182]; Exhibit self-renewal capacity [183].	Carry a risk of rejection or tumor formation [40]; Require appropriate growth media and modulation with transcription factors [150]; Involve ethical controversies that lead to restrictive use, depending on legislation [182].	Modulate the self-renewal properties of ESCs using HA-Tyr hydrogels [184]; Maintain ESCs in an undifferentiated state through encapsulation in HA hydrogels, and HA binding sites influence ESC receptors [185]; Promote the differentiation of ESCs into neural precursor cells using Matrigel [186]; Induce the transformation of ESCs into iPSCs using gelatin gels [169].
iPSCs	Differentiate into a wide range of tissues, including cartilage [158], blood vessels [159], skeletal muscle [161,162], glial cells [163], and skin [164]; Support the development of complex organoids [165,166]; Modulate the ototoxic effects of gentamicin and cisplatin on the inner ear [168]; Treat hearing loss using IEOs derived from iPSCs [170].	Differentiation into specific mature cells through induction with OCT4, SOX2, KLF4 and c-MYC [157,158,159]; Avoid the ethical controversies associated with ESCs [159,160]; Allow for self-assembly into embryoid bodies [187]; Serve as a source for exosomes production [182]; Exhibit anti-fibrotic effects [188].	Carry a risk of malignant transformation during handling [159,160]; Requires adherence to strict regulatory requirements and safety standards for clinical use [187]; Exhibit high variability across methods, resulting in low reproducibility and reliability [187].	Develop IEOs using hydrogels—such as norbornene-functionalized gelatin cross-linked with thiolated PEG, or heparin-functionalized HA—to induce differentiation into ectoderm, mesoderm, and endoderm [169]; Supplement with Matrigel to map the developmental stages of IEOs [172] and produce human neuronal organoids [171].
MSCs	Protect inner ear cells against the ototoxic effects of chemotherapy using MSC-derived EV [177]; Support auricular cartilage regeneration [17]; Promote laryngeal cartilage and vocal cord regeneration [179,180,181].	Possess self-renewal ability [173,174]; Differentiate into organ-specific cell type [173,174]; Diminish ethical concerns compared to ESCs [175]; Reduce the risk of malignant transformation [175]; Serve as a source of exosomes [189]; Reduce inflammation [189].	Exhibit significant heterogeneity, complicated standardization; this is further influenced by donor-to-donor variability, including factors such as species, gender, and health status [190]; Possess limited capacity, perpetual self-renewal, and replication [190]; Show limited potential to differentiate into derivatives of the endoderm, mesoderm, and ectoderm [191].	Facilitate auricular cartilage construction through the interaction of adipose-derived MSCs with type 1 collagen [178]; Promote wound repair and cell proliferation using umbilical cord MSCs-derived exosomes encapsulated in SF/sericin hydrogel dressing [189]; Prolong the therapeutic duration of bone marrow MSCs using alginate hydrogels, which function as reservoirs for sustained release [189].

**Table 2:** Comparative evaluation of bioprinting techniques.

Printing Technology	Advantages	Drawbacks	References
Inkjet bioprinting	High resolution; High throughput; High water content to facilitate nutrient diffusion; Excellent reproducibility; Good cytocompatibility; High cell viability (>90%); Cost-effective; Scalable for multiple printheads.	Direct cellular stress that could lead to membrane damage and irreversible cell alterations; Unsuitable for large-scale constructs; Limited bioink reservoir volume; Incompatible with high viscosity bioinks.	[207,212,213,214,215,221,222,223,224]
Extruded bioprinting	Large-scale tissue engineering; Compatible with a range of materials and viscosities; User-friendly operation.	Direct cellular stress that could compromise viability; Low resolution; Fine structures cannot be accurately reproduced; Filament size is limited by the nozzlediameter.	[207,216,221]
Laser-assisted bioprinting	Minimal cellular stress; Suitable for biolayers or single-layer printing; High cell viability (>90%); High resolution; Precise control ofcell distribution within the bioink.	Prohibitively expensive; Complex setup and maintenance; Cell density below 10^8^ cells/mL.	[207,216,217,223,224]
Stereolithography	High spatial resolution; High precision and high speed printing; Rapid layer-by-layer solidification; High cell viability.	UV-induced cell damage; requires optical transparent materials for light penetration; The photopolymer substrate cannot be changed during the printing process.	[210,221,222]

## Data Availability

No new data were created or analyzed in this study. Data sharing is not applicable to this article.

## Abbreviations

The following abbreviations are used in this manuscript:

AGEs	Advanced glycated end products
CG	Carrageenan
c-MYC	Cellular-myelocytomatosis
DM	Diabetes mellitus
ECM	Extracellular matrix
ENT	Ear, nose, throat
ESC	Embryonic stem cell
EV	Extracellular vesicle
GAG	Glycosaminoglycan
Gal	Galactose
GelMA	Gelatin-methacrylate
GG	Gellan gum
Glc	Glucose
GlcA	Glucuronic acid
GlcNAc	N-acetylated glucosamine
HA	Hyaluronic acid
hNC	Human nasoseptal chondrocytes
hNEC	Human nasal epithelial cell
IEO	Inner ear organoids
KLF4	Kruppel-like factor 4
iPSC	Induced pluripotent stem cell
MMP	Matrix metalloproteinase
MSC	Mesenchymal stem cell
OCT4	Octamer-binding transcription factor 4
PEG	Polyethylene glycol
PEGDA	Polyethylene glycol diacrylate
PNIPAM	Poly-N-isopropylamide
ROS	Reactive oxygen species
SC	Synthetic cell
SF	Silk fibroin
SOX2	Sex determining region Y-box2
TM	Tympanic membrane
